# Deguelin Attenuates Non-Small-Cell Lung Cancer Cell Metastasis by Upregulating PTEN/KLF4/EMT Signaling Pathway

**DOI:** 10.1155/2022/4090346

**Published:** 2022-05-21

**Authors:** Guohua Lu, Yinan Yao, Xiaochen Zhang, Dawei Cui, Jianying Zhou

**Affiliations:** ^1^Department of Respiratory Diseases, The First Affiliated Hospital, Zhejiang University School of Medicine, Hangzhou 310003, China; ^2^Departments of Medical Oncology and Pathology, The First Affiliated Hospital, Zhejiang University School of Medicine, Hangzhou 310003, China; ^3^Department of Blood Transfusion, The First Affiliated Hospital, Zhejiang University School of Medicine, Hangzhou 310003, China

## Abstract

Non-small-cell lung cancer (NSCLC) is the most common lung cancer and a major cause of cancer mortality worldwide. Deguelin plays a vital inhibitory role in NSCLC initiation and development. However, the downstream mechanism of deguelin-suppressed metastasis of NSCLC cells is still not completely understood. Interestingly, phosphatase and tensin homologue deleted on chromosome 10 (PTEN) and Krüppel-like factor 4 (KLF4) also contribute to inhibition of metastasis in NSCLC cells. Here, we demonstrated that deguelin significantly upregulated PTEN and KLF4 expressions and PTEN positively upregulated KLF4 expression in NSCLC cells including A549 and PC9 cells. Moreover, overexpressions of PTEN and KLF4 inhibited the migration and invasion of NSCLC cells, an effect similar to that of deguelin. Furthermore, overexpressions of PTEN and KLF4 could suppress the epithelial-mesenchymal transition (EMT), an effect also similar to that of deguelin. Additionally, deguelin displayed a significant antitumor ability by upregulating PTEN and KLF4 expressions in mice model with NSCLC cells. Together, these results indicated that deguelin could be a potential therapeutic agent through upregulating PTEN and KLF4 expressions for NSCLC therapy.

## 1. Introduction

Lung cancer has become the leading cause of cancer-related deaths worldwide, especially in non-small-cell lung cancer (NSCLC), which accounts for about 85% of all lung cancer cases [1–4]. The burden of lung cancer has become one of the major public health problems in the world. In recent years, various studies on lung cancer and its drugs have made some progress, but the five-year survival rate of patients caused by factors such as adverse drug reactions has not been effectively improved. Thus, the treatment of lung cancer patients is still a big medical problem [3–5].

In recent years, a number of studies have shown that herbal extracts have become a new strategy for the treatment of tumors. For example, the Chinese herbal extract of deguelin, derived from *Lonchocarpus*, *Derris*, or *Tephrosia*, can effectively inhibit the proliferation, invasion, and metastasis of a variety of tumors (e.g., colon cancer, human pancreatic cancer, breast cancer, and lung cancer) [6, 7]. Importantly, deguelin can enhance the sensitivity of tumor cells to chemotherapy drugs and radiotherapy and has no obvious toxicity and inhibitory effect on the growth of normal cells [6]. The main antitumor effects of deguelin include inhibiting the proliferation, invasion, and metastasis of tumor cells; promoting the apoptosis of tumor cells; delaying the tumor cell cycle; and inducing DNA damage of tumor cells [8–13]. However, the molecular mechanisms of deguelin in antitumor effects remain completely unclear, a situation that needs to be explored in the future.

The activation of tumor suppressor genes and oncogenes, including phosphatase and tensin homologue deleted on chromosome 10 (PTEN) and Krüppel-like factor 4 (KLF4), plays a key role in regulating the occurrence and development of tumors. PTEN is a tumor suppressor gene that is closely related to tumorigenesis, and its functional defect exists widely in many kinds of tumors [14–16]. KLF4 plays a dual role in both oncogenes and tumor suppressor genes, and its expression is tissue or cell specific [17–20]. Studies have shown that PTEN and KLF4 are less active in NSCLC, and their high expression can effectively inhibit the proliferation of NSCLC [6, 7, 17, 18]. However, the relationship between PTEN and KLF4 in NSCLC remains unclear, and whether the deguelin affect the expression of PTEN or KLF4 has not been reported.

Numerous studies have shown that epithelial-mesenchymal transition (EMT) of tumor cells plays an important role in tumorigenesis and invasion [21, 22]. In studies of pancreatic cancer, researchers found that deguelin prevented epithelial cells from transforming into mesenchymal cells by inhibiting EMT [8, 23]. Moreover, deguelin inhibited the invasion, metastasis, and EMT transformation of NSCLC, colorectal cancer, and pancreatic tumors [24–26]. Therefore, the inhibition of the EMT process is an important measure in the treatment of tumors. These findings imply that deguelin plays important roles in pathogenesis of the tumors by inhibiting the EMT level. Currently, the accumulated evidence suggests that inactivation or loss of PTEN promotes the poor prognosis and metastasis of cancers by upregulating EMT expression including lung cancer [27, 28]. Similarly, KLF4 can negatively regulate the expression of EMT that is closely associated with the proliferation, invasion, and metastasis of cancer cells including breast cancer and colorectal cancer [29, 30]. These findings imply a possible relation between PTEN and KLF4 in invasion and metastasis of cancer cells that are involved with EMT expression. However, the relation in NSCLC still was completely unclear.

Therefore, this study was done to analyze the regulation of PTEN and KLF4 expressions in NSCLC cells by deguelin in vitro and in mice and to improve the mechanism of deguelin inhibiting the proliferation of NSCLC to explore the potential value of deguelin in the treatment of NSCLC.

## 2. Materials and Methods

### 2.1. Cell Culture

Human lung cancer cell lines A549 and PC9, purchased from the Committee on Type Culture Collection of Chinese Academy of Sciences (Shanghai, China), were cultured in PRMI-1640 medium, containing 10% fetal bovine serum (FBS), 100 U penicillin, and 100 *μ*g streptomycin, and then, the cells were cultured in cell incubator at 37°C with 5% CO_2_.

### 2.2. Quantitative Real-Time PCR

Total cellular RNA was extracted by RNeasy Mini Kit (74106, Qiagen, Germany), based on the manufacturer's protocol, the concentration of which was measured by NanoDrop 2000 (Thermo Scientific, USA). The total RNA was reverse transcribed into complementary DNA (cDNA) by PrimeScript 1st Strand cDNA Synthesis Kit (D6110A, Takara, China); then, real-time quantitative polymer chain reaction (qPCR) for cDNA amplification was carried out by QuantiFast SYBR Green PCR Kit (Qiagen, Germany). The relative levels of messenger RNA (mRNA) expression were calculated by the comparative Ct method (2^-*ΔΔ*Ct^). The glyceraldehyde 3-phosphate dehydrogenase (GAPDH) was considered as an internal control of gene expression [8]. The specific primers of real-time qPCR are shown in Table 1.

### 2.3. Immunoblotting

Immunoblotting was performed as previously described [8]. Briefly, the cells were washed twice using cold phosphate buffered saline (PBS) and lysed with lysis buffer, supplied with protease and phosphatase inhibitors, at 4°C for 30 min. The lysate supernatants were harvested and boiled in loading buffer. Protein concentration was tested by Pierce BCA Protein Assay Kit (23227, Thermo Scientific, USA). Cell lysates were followed by SDS–PAGE gel electrophoresis and then transferred to polyvinylidene fluoride (PVDF) membrane (Millipore, USA) for immunoblotting analysis and antibody hybridization. The target protein bands were visualized by an enhanced chemiluminescence system (Bio-Rad, California, USA). The antibodies PTEN (9559), KLF4 (4038), Claudin-1 (4933), Cyclin D1 (2978), E-cadherin (3195), N-cadherin (13116), survivin (71G4B7), Vimentin (Cat#5741), and *β*-actin (3700) were obtained from Cell Signaling Technology (Danvers, MA, USA).

### 2.4. Cell Transfection

NSCLC cells were transfected with PTEN or KLF4 small interfering RNA (siRNA) sequences (or overexpression plasmids) or negative control, purchased from RiboBio (Guangzhou, China), by Lipofectamine 2000 (11668019, Invitrogen, USA), according to the protocols, to determine PTEN or KLF4 knockdown (or overexpression) in the two cell lines, respectively. After transfection, the cells were collected for further experimentation. The transfection efficiency was confirmed by immunoblotting to analyze the expression levels of PTEN or KLF4 protein.

### 2.5. Cell Scratch Assay

A cell scratch assay was performed to evaluate cell motility. The transfected cells were seeded and cultured in six-well plates. The wound healing was scratched by a 100 *μ*L sterile pipette tip, and the cells were washed three times with PBS. The wound healing width was observed in five different areas at 48 h by an inversion fluorescence microscope (Olympus, Japan).

### 2.6. Cell Invasion Assay

The invasion experiment was carried out in a transwell. The NSCLC cells in a serum-free medium were inoculated into the upper chamber of the transwell, and the 24-well plate in the lower chamber was filled with RPMI 1640 culture medium. The cells in the upper chamber were wiped out after 48 h, and those in the lower chamber were stained with 1% crystal violet. The chamber was precoated with Matrigel (BD Bioscience, USA) to evaluate cell invasion. The cells were counted in at least three random fields.

### 2.7. Animal Experiments

Six-week-old female BALB/c-nude mice were obtained from Shanghai Experimental Animal Center (Chinese Academy of Sciences, China) for human tumor models. After two-week acclimatization, they were randomized into groups of six mice. The control group was injected with 2 × 10^6^/cells with PC9 cells per mouse. The experimental group were injected with an equal number of PC9 cells. When palpable tumors (~50-100 mm^3^) arose, the control group was orally treated with physiological saline, and experimental group were treated with deguelin (4 mg/kg) by oral gavage on 1, 3, and 5 days of each week for three weeks. Tumor size was tested by caliper through measurements of the two perpendicular diameters every three days using the formula: Tumor Volume = (width^2^ × length)/2. All procedures were performed according to the Regulations for the Administration of Affairs Concerning Experimental Animals. The experiments were approved by the Experimental Animal Ethics Committee of the First Affiliated Hospital, Zhejiang University School of Medicine.

### 2.8. Statistical Analysis

The experimental data were analyzed by GraphPad 6.04 software (GraphPad Software Inc., La Jolla, USA). All the experiments were independently repeated three times. The results were expressed by mean ± standard deviation (SD), *t*-test, and analysis of variance between groups of samples; *P* value < 0.05 was considered to be statistically significant.

## 3. Results

### 3.1. Deguelin Upregulates the Expressions of PTEN and KLF4

**Figure 1:**
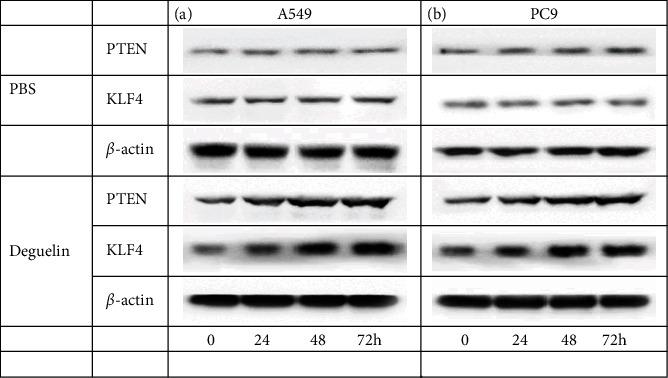
Deguelin upregulates PTEN and KLF4 expression in NSCLC cells. (a) The expression levels of PTEN and KLF4 in A549 cells induced by deguelin (25 *μ*M) at different times compared to controls treated by PBS. (b) The expression levels of PTEN and KLF4 in PC9 cells induced by deguelin at different times compared to controls treated by PBS. *β*-Actin was used as an internal control. All experiments were repeated at least in triplicate.

To investigate the effect of deguelin on the expressions of PTEN and KLF4, we added 25 *μ*mol (*μ*M) deguelin into A549 and PC9 cells and detected the changes of PTEN and KLF4 proteins by immunoblotting assay at different time points (0, 24, 48, and 72 h). The results showed that compared to controls, deguelin significantly upregulated the expressions of PTEN and KLF4 in a time-dependent manner in A549 cells and PC9 cells, respectively (Figures 1(a) and 1(b)). Moreover, significant differences of PTEN and KLF4 gene expressions were observed at 48 h by PCR assay (Fig. 1S).

### 3.2. Determination of Time Point of Target Gene Overexpression and Interference Experiment

**Figure 2:**
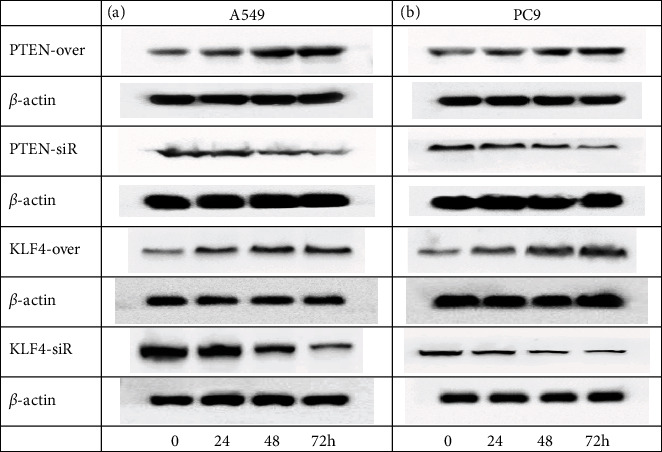
Determination of PTEN-siR/overexpression and KLF4-siR/overexpression in NSCLC cells at different times. (a) The expression levels of PTEN and KLF4 in A549 cells treated by PTEN-siR/overexpression and KLF4-siR/overexpression at different times compared to controls treated by PBS, respectively. (b) The expression levels of PTEN and KLF4 in PC9 cells treated by PTEN-siR/overexpression and KLF4-siR/overexpression at different times compared to controls treated by PBS, respectively. *β*-Actin was used as an internal control. All experiments were repeated at least in triplicate.

To determinate the appropriate time point of target gene expression, we determined the time point (0, 24, 48, and 72 h) of overexpression and siRNA in the experiment on PTEN and KLF4 genes in A549 cells and PC9 cells to facilitate the follow-up experiment. The best time point for overexpression and siRNA of PTEN and KLF4 genes was 48 h in A549 cells and PC9 cells, respectively (Figures 2(a) and 2(b)). Moreover, significant changes of PTEN and KLF4 gene expressions were observed at 48 h by PCR assay (Fig. 1S).

### 3.3. Deguelin Inhibits the Migration and Invasion of NSCLC Cells

**Figure 3:**
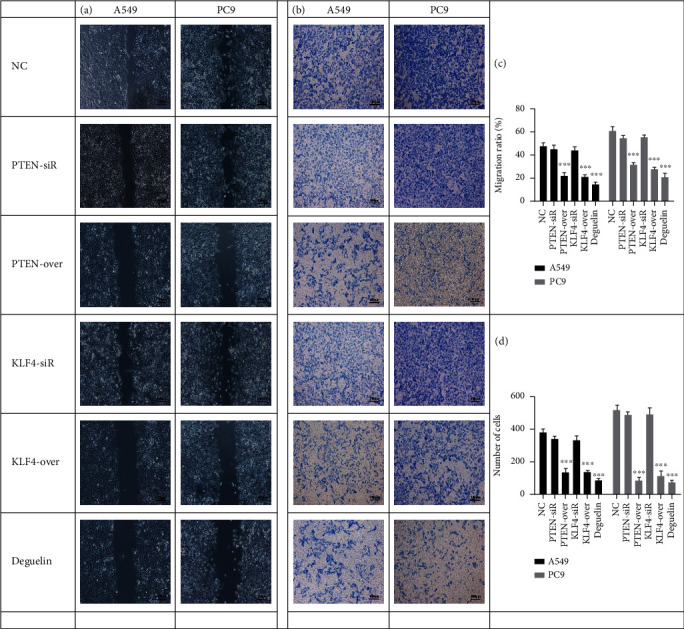
Deguelin inhibits migration and invasion of NSCLC. (a and c) The cell scratch assay for A549 and PC9 cell migration after cells were transfected by PTEN-siR/overexpression, KLF4-siR/overexpression, and deguelin (25 *μ*M), respectively. The migration area was counted. (b and d) The cell invasion assay for A549 and PC9 cells after cells were transfected by PTEN-siR/overexpression, KLF4-siR/overexpression, and deguelin (25 *μ*M), respectively. The numbers of invasion cells were counted. Scale bar represents 100 *μ*m. All experiments were repeated at least in triplicate. The data are presented as the mean ± SD. Significant differences are indicated by ^∗∗∗^*P* < 0.001.

To investigate the effect of deguelin on the migration and invasion of NSCLC cells, we added deguelin (25 *μ*M) into A549 and PC9 cells for 48 h. The results of the scratch analysis showed that deguelin could effectively inhibit the migration of A549 and PC9 cells, the migration effect of PTEN and KLF4 induced by their overexpression was similar to that of deguelin, and the effect on siRNA of PTEN and KLF4 was similar to that of normal control (NC) but contrary to the effect on deguelin (Figures 3(a) and 3(c)). The invasive effect of PTEN and KLF4 on NSCLC cells was similar to that of deguelin, and the effect on siRNA of PTEN and KLF4 in NSCLC cells was similar to that of NC but different from that of deguelin (Figures 3(b) and 3(d)).

### 3.4. Relationship between PTEN and KLF4

**Figure 4:**
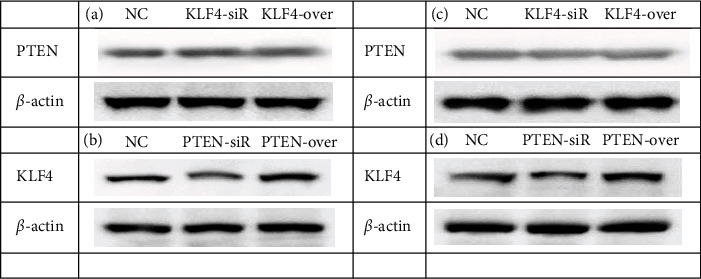
PTEN upregulates KLF4 expression in NSCLC cells. (a) The expression levels of PTEN in A549 cells transfected with KLF4-siR/overexpression were detected by immunoblotting assay. (b) The expression levels of KLF4 in A549 cells transfected with PTEN-siR/overexpression were detected by immunoblotting assay. (c) The expression levels of PTEN in PC9 cells transfected with KLF4-siR/overexpression were detected by immunoblotting assay. (d) The expression levels of KLF4 in PC9 cells transfected with PTEN-siR/overexpression were detected by immunoblotting assay. *β*-Actin was used as an internal control. All experiments were repeated at least in triplicate.

To determine whether PTEN affects the expression of KLF4 in NSCLC cells, siRNA and overexpressions of PTEN and KLF4 were analyzed in this study. The results showed that the overexpression or siRNA of KLF4 had no significant effect on PTEN expression in A549 cells and PC9 cells (Figures 4(a) and 4(c)). Interestingly, both overexpression and siRNA of PTEN positively regulated KLF4 expression in A549 cells and PC9 cells (Figures 4(b) and 4(d)).

### 3.5. Effect of Deguelin on EMT Expression by PTEN and KLF4

**Figure 5:**
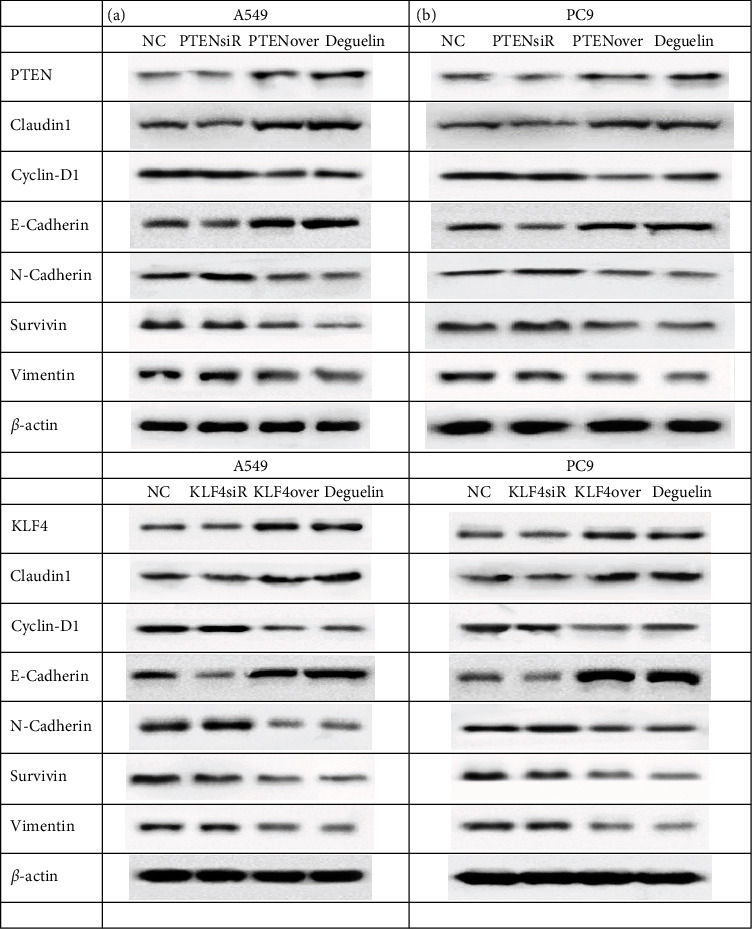
EMT-associated proteins were regulated by deguelin, PTEN, and KLF4. (a) EMT-associated proteins were detected in A549 cells and PC9 cells by deguelin and PTEN-siR/overexpression. (b) EMT-associated proteins were detected in A549 cells and PC9 cells by deguelin and KLF4-siR/overexpression. *β*-Actin was used as an internal control. All experiments were repeated at least in triplicate.

To determine the effect of deguelin, PTEN, and KLF4 on EMT in NSCLC cells, deguelin, siRNA, and overexpressions of PTEN and KLF4 were analyzed in this study. The results showed that deguelin can effectively inhibit EMT in A549 cells and PC9 cells by decreasing vimentin protein expression and promoting E-cadherin level, an effect that was similar to that of PTEN and KLF4 overexpressions on the EMT in A549 cells and PC9 cells (Figures 5(a) and 5(b)). However, the effect of deguelin was different from that of PTEN and KLF4 siRNA, which promotes EMT expressions in A549 cells and PC9 cells (Figures 5(c) and 5(d)).

### 3.6. Effect of Deguelin on Tumor Size in Tumor-Bearing Mice

**Figure 6:**
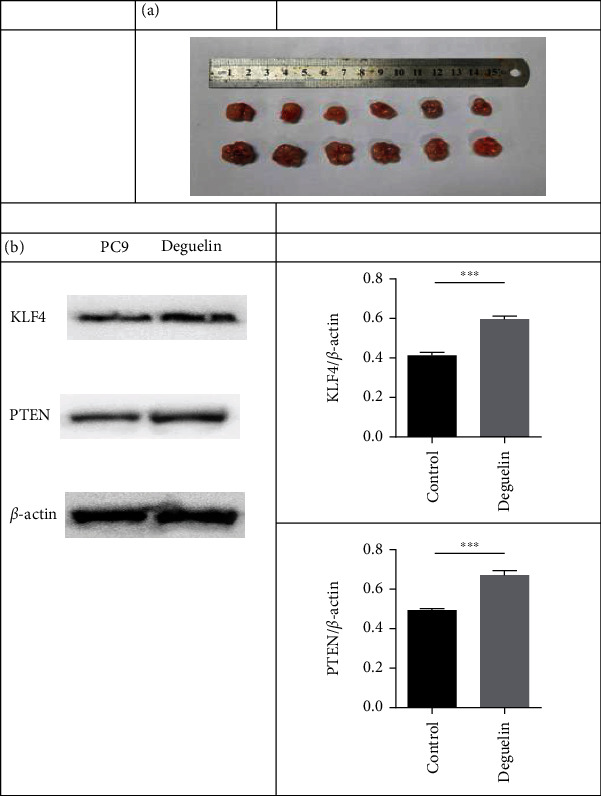
Deguelin inhibited tumor growth by upregulating PTEN and KLF4 expressions in mice model with NSCLC cells. (a) On day 21, the tumors were carefully dissected from the mice, and the tumor size was measured. (b) The expression levels of KLF4 and PTEN proteins in tumors were detected. (c and d) The relative band intensity of KLF4 and PTEN in tumors were detected. *β*-Actin was used as an internal control. All experiments were repeated at least in triplicate. ^∗∗∗^*P* < 0.001 and ^∗∗∗∗^*P* < 0.0001.

To further analyze the effect of deguelin on tumor size and expressions of PTEN and KLF4, we inoculated PC9 cells subcutaneously into BABL/c mice and injected deguelin. After 2 weeks, the tumor size was measured, and the expressions of PTEN and KLF4 were detected. The results showed that deguelin could effectively inhibit tumor growth and upregulate the expressions of PTEN and KLF4 in tumor tissues (Figure 6).

## 4. Discussion

NSCLC is the most common lung cancer and is a major cause of cancer-related deaths worldwide. The metastasis of NSCLC is the key factor for its poor prognosis [3–5, 31]. The burden of disease with NSCLC has become one of the major public health problems in the world. Here, we observed that deguelin could significantly upregulate PTEN and KLF4 expressions in NSCLC cells, including A549 and PC9 cells in this study. Interestingly, PTEN could positively upregulate KLF4 expression in A549 and PC9 cells. Moreover, overexpressions of PTEN and KLF4 or deguelin could inhibit the migration and invasion of NSCLC cells, which were involved into EMT expressions in NSCLC cells. Additionally, deguelin displayed a significant antitumor ability by upregulating PTEN and KLF4 expressions in mice model with NSCLC cells. Together, these results indicated that deguelin was considered to be a potential therapeutic target for the treatment of NSCLC.

In recent years, deguelin, a rotenoid of the flavonoid family, extracted from *Lonchocarpus*, *Derris*, or *Tephrosia*, can effectively inhibit the proliferation, invasion, and metastasis of many kinds of tumors, including colon cancer, human pancreatic cancer, breast cancer, and lung cancer, and is a promising chemopreventive agent for cancer therapy [6, 7]. Deguelin promotes apoptosis of NSCLC by inhibiting galectin-1 protein expression [8]. Deguelin derivatives block the development of NSCLC by interfering with the binding of adenosine triphosphate (ATP) to heat shock protein 90 (HSP90); its analogue SH-1242 also exerts its antitumor effect by inhibiting HSP90 [32, 33]. Researchers found that deguelin could inhibit the proliferation, invasion, metastasis, and autophagy of tumor cells by regulating many signal pathways (e.g., EGFR/IGF1R-Akt, MAPK, and mTOR). Our study also confirmed that deguelin can effectively inhibit the invasion, migration, and growth of NSCLC cells [6–10, 34, 35]. In the xenograft mouse model, orally treated with deguelin (4 mg/kg/three times a week) significantly prevented tumor growth, according to the dose conversion [36], the 4 mg/kg deguelin dose used in mouse is equivalent to the dose of 19.5 mg deguelin dose for a 60 kg person, which is certainly within the range of a number of plant extracts. These data provide a strong basis for the future clinical translational research of deguelin.

Studies show that PTEN, as a tumor suppressor gene, its functional defect plays a key role in the development of various cancers, including prostate cancer, lung cancer, hepatocellular carcinoma (HCC), and pancreatic cancer [14, 37–40]. The low expression or loss of function of PTEN in patients and animal models with NSCLC could not effectively inhibit the proliferation and migration of NSCLC [41–43]. These results indicated that increased PTEN will contribute to inhibition of tumors. Our study also showed that deguelin could effectively promote the expression of PTEN to inhibit the invasion and migration of NSCLC cells in vitro, which was associated with inhibition of EMT, and suppressed tumor growth with upregulation of PTEN expression in tumor tissue from mice model with NSCLC. These findings further confirmed the important value of deguelin as an antitumor agent for NSCLC by upregulating PTEN expression to decrease the EMT.

Previous reports showed that KLF4, a zinc finger-type transcription factor, played a pivotal and different role in the development of various cancers, including lung cancer, HCC, and pancreatic cancer [44–48]. However, low expression of KLF4 promoted the growth, invasion, and metastasis of NSCLC, but high expression of KLF4 displayed a valuable role for therapy of NSCLC [49–51]. In our study, the results showed that increased expression of KLF4 could effectively inhibit the growth, invasion, and metastasis of NSCLC, which was associated with inhibition of EMT in cell lines. Interestingly, deguelin could significantly promote the expression of KLF4 in cell lines and mouse tumor tissue of NSCLC to play an important antitumor role. These findings indicated that deguelin could effectively suppress the growth, invasion, and metastasis of NSCLC by upregulating KLF4 expression to reduce the EMT. Importantly, deguelin could suppress the invasion and metastasis of NSCLC by upregulating PTEN and KLF4 expressions to reduce the EMT, which indicated an important relation between PTEN and KLF4 in NSCLC. Our results demonstrated the value of the hypothesis about the relation between PTEN and KLF4 in NSCLC. Overexpressed PTEN could promote KLF4 expression to inhibit the EMT, and siPTEN attenuated KLF4 expression to restore the EMT. However, overexpressed KLF4 (or siKLF4) could not enhance (or decrease) PTEN expression but could affect the EMT in NSCLC cell lines. These findings confirmed that PTEN could promote KLF4 expression to suppress EMT in NSCLC and deguelin is a promising agent for NSCLC therapy. However, how does deguelin regulate the PTEN/KLF4/EMT process is explored in the future study.

## 5. Conclusion

In summary, deguelin effectively promoted the expression of PTEN and KLF4 in NSCLC cells in vitro, and upregulated PTEN could increase the expression of KLF4 to suppress the EMT to further attenuate the invasion and migration of NSCLC cells. In vivo experiments also showed that deguelin could upregulate the expression of PTEN and KLF4 in tumor-bearing mice and then significantly inhibit the growth of NSCLC in mice. These findings further improved the important molecular mechanism of deguelin inhibiting the invasion and migration of NSCLC and established an important foundation for exploring the potential value of deguelin as a promising drug for NSCLC therapy.

## Figures and Tables

**Table 1:** Specific primers used for real-time PCR in this study.

Primer name	Sequences (5′ to 3′)
PTEN-F	5-TGGATTCGACTTAGACTTGACCT-3
PTEN-R	5-GGTGGGTTATGGTCTTCAAAAGG-3
KLF4-F	5-TCGGACCACCTCGCCTTACA-3
KLF4-R	5-TCGGACCACCTCGCCTTACA-3
GAPDH-F	5-GGAGCGAGATCCCTCCAAAAT-3
GAPDH-R	5-GGCTGTTGTCATACTTCTCATGG-3

## Data Availability

The data used to support the findings of this study are included within the article.

## Supplementary Materials

Supplementary Materials Fig. 1S: expression levels of PTEN and KLF4 mRNAs in NSCLC cells at 48 h. (A and B) The relative levels of PTEN mRNA in A549 cells and PC9 cells treated with deguelin and transfected with PTEN-siR/overexpression. (C and D) The relative levels of KLF4 mRNA in A549 cells and PC9 cells treated with deguelin and transfected with PTEN-siR/overexpression. GAPDH was used as an internal control. All experiments were repeated at least in triplicate. ^∗^*P* < 0.05, ^∗∗^*P* < 0.01, and ^∗∗∗^*P* < 0.001.
