# Supplementary material for: Deguelin Attenuates Non-Small-Cell Lung Cancer Cell Metastasis by Upregulating PTEN/KLF4/EMT Signaling Pathway
Source: Dis Markers. 2022 May 21;2022:4090346. doi: 10.1155/2022/4090346 (PMC9148257; doi:10.1155/2022/4090346)
Supplement: Supplementary Materials — Fig. 1S: expression levels of PTEN and KLF4 mRNAs in NSCLC cells at 48 h. (A and B) The relative levels of PTEN mRNA in A549 cells and PC9 cells treated with deguelin and transfected with PTEN-siR/overexpression. (C and D) The relative levels of KLF4 mRNA in A549 cells and PC9 cells treated with deguelin and transfected with PTEN-siR/overexpression. GAPDH was used as an internal control. All experiments were repeated at least in triplicate. ∗P < 0.05, ∗∗P < 0.01, and ∗∗∗P < 0.001. [file 4090346.f1.docx]

**Fig. 1S**

| **A A549** | **B PC9** |
| --- | --- |
| 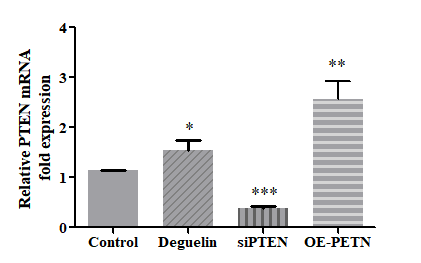 | 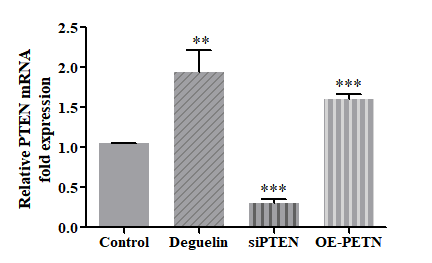 |
| **C** | **D** |
| 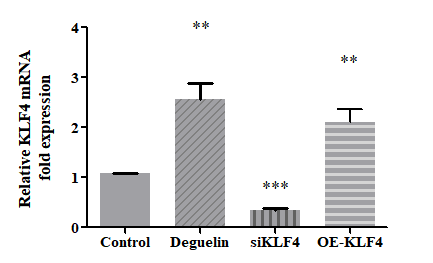 | 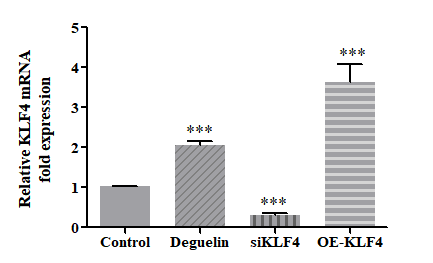 |
| **Fig. 1S. Expression levels of PTEN and KLF4 mRNAs in NSCLC cells at 48 h.** (A-B) The relative levels of PTEN mRNA in A549 cells and PC9 cells treated with deguelin and transfected with PTEN-siR/overexpression. (C-D) The relative levels of KLF4 mRNA in A549 cells and PC9 cells treated with deguelin and transfected with PTEN-siR/overexpression. GAPDH was used as an internal control. All experiments were repeated at least in triplicate. **P*<0.05, ***P*<0.01, ****P*<0.001. |  |
